# Revisiting midwifery's identity: A crucial step to support access to continuity models of care

**DOI:** 10.18332/ejm/205283

**Published:** 2025-06-25

**Authors:** Josyane Giroux

**Affiliations:** 1 Capitale-Nationale Birth Center, CIUSSS de la Capitale-Nationale, Québec, Canada; 2 Midwifery, Faculty of Health Sciences, McMaster University, Hamilton, Canada

**Keywords:** midwifery, health care systems, sexual and reproductive health rights

Globally, midwifery is at a pivotal moment. Leading international health organizations, including the World Health Organization (WHO), the United Nations Population Fund (UNFPA), and the International Confederation of Midwives (ICM), have recently published global position papers outlining the vital role and impact that midwives can have within healthcare systems, as well as the importance of investing in midwifery-led models of care^1-3^. In response to these calls to action, many countries are working to expand access to midwifery care.

However, implementing these changes within existing systems may lead to confrontations, marginalization, and a dilution of midwifery’s core philosophy and values. This should encourage midwives to come together and develop a collective sense of their profession and a shared vision. In the same vein as Gagnon and Lemay^4^, finding the most suitable words to describe how midwives navigate risks, uncertainties, and varied spaces, from sacred to medical, is essential to support the three pillars of the profession – educators, associations, and regulators – in defending autonomy and advancing the profession.

In Canada, more than thirty years after its gradual legal recognition across the different provinces, midwives still navigate tensions between their community-based and person-centered roots and the institutional, biomedical and colonial systems in which they are required to practice. The profession was legalized based on the continuity model of care in response to pregnant individuals' reclamation of de-medicalization of childbirth and autonomy in perinatal care. In contrast with the international endorsement of midwifery-led continuity models, the sustainability of the Canadian model of care is under scrutiny by professional associations, research teams and governments^2,5,6^.

Midwives across the country are reporting high burnout and attrition rates, which threaten workforce balance and equitable access to care^6^. These challenges are complex and systemic, and addressing them requires structural adjustments and a broader cultural and organizational transformation^6,7^. Policies must be adapted to reflect what matters to midwives and their clients rather than what fits the systems^8^. To respond meaningfully, we must deepen our understanding of midwifery's unique paradigm and professional values. This includes engaging midwives and the communities they serve in dialogue on what defines the midwives' identity.

Throughout my fifteen years of involvement in midwifery activism – particularly as former president of the *Regroupement Les sages-femmes du Québec* and a board member of the Canadian Association of Midwives – I have come to realize that clarifying and affirming our professional identity and articulating our distinct contributions at the local level is critical to resisting systemic pressures. This involves a deeper understanding of our societal mission, the necessary advocacy work, and the systems in which midwives are involved. In Quebec, the results of the Gagnon and Lemay^4^ research project shape the profession's future and help ensure that innovative models of care and activities remain rooted in the specificity of the practice. Sustainability must resonate with a deep connection to why, how, and for whom we want to do this work individually and collectively.

We invite midwives to undertake the overlooked yet essential task of articulating their local professional identity and reaffirming their specificity within the healthcare system and society. As seen in Canadian provinces and many countries, the dominant biomedical system can influence the profession in ways that erode midwives’ deep sense of purpose and midwifery’s capacity for systemic change. In today's global context, where reproductive rights are threatened and healthcare is increasingly fragmented, with an emphasis on risk management and performance indicators, engaging in this work through participatory research or other collaborative methods, is a recognition of midwives' distinctive contribution to advancing reproductive and sexual health rights. Furthermore, it is a collective act of care.

## Data Availability

Data sharing is not applicable to this article as no new data were created.
